# COVID-19 in unvaccinated patients with inborn errors of immunity—polish experience

**DOI:** 10.3389/fimmu.2022.953700

**Published:** 2022-09-23

**Authors:** Sylwia Kołtan, Marcin Ziętkiewicz, Elżbieta Grześk, Rafał Becht, Elżbieta Berdej-Szczot, Magdalena Cienkusz, Marlena Ewertowska, Edyta Heropolitańska-Pliszka, Natalia Krysiak, Aleksandra Lewandowicz-Uszyńska, Monika Mach-Tomalska, Aleksandra Matyja-Bednarczyk, Marcin Milchert, Katarzyna Napiórkowska-Baran, Karolina Pieniawska-Śmiech, Anna Pituch-Noworolska, Joanna Renke, Jacek Roliński, Iwona Rywczak, Agnieszka Stelmach-Gołdyś, Magdalena Strach, Hanna Suchanek, Joanna Sulicka-Grodzicka, Aleksandra Szczawińska-Popłonyk, Sławomir Tokarski, Ewa Więsik-Szewczyk, Beata Wolska-Kuśnierz, Krzysztof Zeman, Małgorzata Pac

**Affiliations:** ^1^ Department of Pediatrics, Hematology and Oncology, Collegium Medicum Bydgoszcz, Nicolaus Copernicus University Toruń, Bydgoszcz, Poland; ^2^ Department of Internal Medicine, Connective Tissue Diseases and Geriatrics, Medical University of Gdansk, Gdansk, Poland; ^3^ Clinical Department of Oncology, Chemotherapy and Cancer Immunotherapy, Pomeranian Medical University in Szczecin, Szczecin, Poland; ^4^ Department of Paediatric and Paediatric Endocrinology, Upper-Silesian Paediatric Health Center, Katowice, Poland; ^5^ Department of Paediatric Haematology and Oncology and Transplantology Medical University of Lublin, Lublin, Poland; ^6^ Department of Immunology, Children’s Memorial Health Institute, Warsaw, Poland; ^7^ Department of Pediatrics, Immunology and Nephrology, Institute of Polish Mother’s Health Center, Łódź, Poland; ^8^ 3^rd^ Department and Clinic of Paediatrics, Immunology and Rheumatology of Developmental Age, Wroclaw Medical University, Wroclaw, Poland; ^9^ Department of Clinical Immunology and Paediatrics, Provincial Hospital J. Gromkowski, Wroclaw, Poland; ^10^ Department of Immunology, University Children’s Hospital of Cracow, Cracow, Poland; ^11^ 2^nd^ Department of Internal Medicine, Jagiellonian University Medical College, Krakow, Poland; ^12^ Department of Internal Medicine Rheumatology Diabetology Geriatrics and Clinical Immunology, Pomeranian Medical University in Szczecin, Szczecin, Poland; ^13^ Department and Clinic of Allergology, Clinical Immunology and Internal Diseases, Ludwik Rydygier Collegium Medicum in Bydgoszcz Nicolaus Copernicus University in Toruń, Bydgoszcz, Poland; ^14^ Department of Clinical Immunology, Wroclaw Medical University, Wroclaw, Poland; ^15^ Department of Pediatrics, Hematology and Oncology, Medical University of Gdańsk, Gdańsk, Poland; ^16^ Department of Clinical Immunology, Medical University of Lublin, Lublin, Poland; ^17^ Department of Hematology, Holycross Cancer Center, Kielce, Poland; ^18^ Department of Immunology, Faculty of Health Sciences, Jan Kochanowski University, Kielce, Poland; ^19^ Department of Rheumatology and Immunology, Jagiellonian University Collegium Medicum, Kraków, Poland; ^20^ Department of Pediatric Pneumonology, Allergy and Clinical Immunology, Institute of Pediatrics, Poznań University of Medical Sciences, Poznań, Poland; ^21^ Institute of Medical Sciences, Medical College of Rzeszow University, Rzeszow, Poland; ^22^ Department of Internal Medicine, Pneumonology, Allergology and Clinical Immunology, Central Clinical Hospital of the Ministry of National Defense, Military Institute of Medicine, Warsaw, Poland

**Keywords:** inborn errors of immunity, adults, children, COVID-19 unvaccinated patients, COVID-19

## Abstract

At the beginning of the Severe Acute Respiratory Syndrome Coronavirus 2 (SARS-CoV-2) pandemic, patients with inborn errors of immunity (IEI) appeared to be particularly vulnerable to a severe course of the disease. It quickly turned out that only some IEI groups are associated with a high risk of severe infection. However, data on the course of Coronavirus Disease 2019 (COVID-19) in patients with IEI are still insufficient, especially in children; hence, further analyses are required. The retrospective study included 155 unvaccinated people with IEI: 105 children and 50 adults (67.7% and 32.3%, respectively). Male patients dominated in the study group (94 people, 60.6%). At least two comorbidities were found in 50 patients (32.3%), significantly more often in adults (56% vs. 21%). Adult patients presented significantly more COVID-19 symptoms. Asymptomatic and mildly symptomatic course of COVID-19 was demonstrated in 74.8% of the entire group, significantly more often in children (88.6% vs. 46%). Moderate and severe courses dominated in adults (54% vs. 11.4%). Systemic antibiotic therapy was used the most frequently, especially in adults (60% vs. 14.3%). COVID-19-specific therapy was used almost exclusively in adults. In the whole group, complications occurred in 14.2% of patients, significantly more often in adults (30% vs. 6.7%). In the pediatric group, there were two cases (1.9%) of multisystem inflammatory syndrome in children. Deaths were reported only in the adult population and accounted for 3.9% of the entire study group. The death rate for all adults was 12%, 15.4% for adults diagnosed with common variable immunodeficiency, 12.5% for those with X-linked agammaglobulinemia, and 21.4% for patients with comorbidity. The results of our study imply that vaccinations against COVID-19 should be recommended both for children and adults with IEI. Postexposure prophylaxis and early antiviral and anti-SARS-CoV-2 antibody-based therapies should be considered in adults with IEI, especially in those with severe humoral immune deficiencies and comorbidity.

## Introduction

The first reports from China about the infection caused by the new SARS-CoV-2 virus came in November 2019. On 4 March 2020, the first case of COVID-19 was diagnosed in Poland. On March 11, 2020, The World Health Organization declared COVID-19 a pandemic. From that moment, infections caused by variants of the virus spread around the world, infecting over 527 million people and killing over 6.3 million people (1).

From the outset of the pandemic, patients with inborn errors of immunity (IEI) were identified as particularly vulnerable to SARS-CoV-2 infection and severe COVID-19. However, with emerging reports on the course of the infection in this particular group, it becomes clear that only certain IEI defects are associated with a poor prognosis (2–4). Similarly as in the entire population, the risk of death increases with age and the incidence of comorbidity diseases (5).

In the studies on patients with IEI presented so far, there is a significant difference in the demonstrated risk of a severe course and death due to COVID-19. This seems to be related to a different spectrum of diagnoses of IEI depending on geographic location and cultural differences (especially family relationship between parents) in certain countries (6, 7).

Preventive vaccinations have been and still are the hope for overcoming the pandemic and returning to normal life. The first vaccinations in Poland, in the so-called priority groups, began in December 2020. Patients with IEI were not included in the first place in the group eligible for vaccination. They received the opportunity to be vaccinated against SARS-CoV-2 only in May 2021.

Due to the still-limited data on the course of COVID-19 in children and adults with IEI, it is necessary to collect clinical data to identify IEI-related risk factors for the serious course of COVID-19, as well as to define possible long-term or delayed complications of the infection. This knowledge should translate into the optimization of disease treatment in this specific group of patients. It will also be a reference point for assessing a possible change in the disease pattern in vaccinated patients with IEI. Therefore, the aim of the study was to conduct a clinical analysis of Polish patients diagnosed with IEI and COVID-19, who fell ill prior to the implementation of preventive vaccinations in this group.

## Material

The retrospective study included 155 people: 105 children (67.7%) and 50 adults (32.3%) diagnosed with a disease/syndrome recognized as IEI according to the guidelines of the European Society for Immunodeficiencies (ESID). The characteristics of the study group, the type of IEI, treatment, and comorbidities are presented in the Results section and **Table 1**. 

**Table 1:** Characteristics of the study group.

Feature	Whole group N (%)	Children N (%)	Adults N (%)	p-value
	155 (100.0%)	105 (67.7%)	50 (32.3%)	
**Sex:**				
M/F	94(60.6%)/61(39.4%)	67(63.8%)/38(36.2%)	27(54%)/23(46.0%)	0.292
**Age at diagnosis of IEI:**				
Median [min–max]	6 [0.1–81]	3 [0.1–16]	37 [1–81]	**<0.001**
**Observation period from IEI diagnosis to COVID-19 diagnosis:**				
Median [min–max]	4 [0–34]	3 [0–14]	7 [0–34]	**<0.001**
**Diagnosis:**				
PAD* CVID XLA Other humoral	103/155 (66.5%) 39/155 (25.2%) 10/155 (6.5%) 54/155 (34.8%)	56/105 (53.3%) 13/105 (12.4%) 2/105 (1.9%) 41/105 (39.0%)	47/50 (94.0%) 26/50 (52.0%) 8/50 (16.0%) 13/50 (26.0%)	**<0.001** **<0.001** **0.002** 0.149
CID *(including syndromic)* and SCID**	27/155 (17.4%)	26/105 (24.8%)	1/50 (2.0%)	**<0.001**
Autoinflammatory syndrome***	13/155 (8.4%)	13/105 (12.4%)	0/50 (0%)	**0.01**
Other IEI****	12/155 (7.7%)	10/105 (9.5%)	2/50 (4.0%)	0.339
**Comorbidities:**				
At least 1 ≥ 2	79/155 (51.0%) 49/155 (31.6%)	44/105 (41.9%) 21/105 (20.0%)	35/50 (70.0%) 28/50 (56.0%)	**0.001** **<0.001**
**Treatment of IEI/comorbidities:**				
IgRT HCT Biologicals Immunosuppressants	105/155 (67.7%) 6/155 (3.9%) 6/155 (3.9%) 21/155 (13.5%)	62/105 (59.0%) 6/105 (5.7%) 6/105 (5.7%) 18/105 (17.1%)	43/50 (86.0%) 0/50 (0.0%) 0/50 (0.0%) 3/50 (6.0%)	**<0.001** 0.178 0.178 0.078

CVID, common variable immunodeficiency; CID, combined immunodeficiency; PAD, predominantly antibody deficiency; SCID, severe combined immunodeficiency; XLA, X-linked agammaglobulinemia; IgRT, immunoglobulin replacement therapy; HCT, hematopoietic cell transplantation.*PAD (in children and in adults, respectively): selective IgA deficiency (sIgAD): 6 (3 + 3), isolated IgG subclass deficiency with normal Ig levels and normal B cells: 10 (7 + 3), selective IgM deficiency: 2 (2 + 0), specific antibody deficiency with normal Ig levels and normal B cells: 1 (1 + 0), transient hypogammaglobulinemia of infancy: 3 (3 + 0), severe hypogammaglobulinemia with normal B cells and secondary causes excluded: 32 (25 + 7). **SCID (in children and in adults, respectively): 4 (4 + 0), all T-B+CID (in children and in adults, respectively): Nijmegen breakage syndrome: 6 (6 + 0), DiGeorge syndrome: 4 (4 + 0), Wiskott–Aldrich syndrome: 3 (2 + 1), ataxia–telangiectasia syndrome: 2 (2 + 0), other single cases: 5 (4 + 0), 3 under genetic diagnosis (3 + 0). ***Autoinflammatory disorders only in children: periodic fever aphthous stomatitis, pharyngitis, and adenopathy (PFAPA): 4 (4 + 0),TNF receptor-associated periodic syndrome, familial cold autoinflammatory syndrome, mevalonate kinase deficiency: one case eachUnclassified autoinflammatory disorders undergoing genetic diagnosis: 6. ****other IEI (in children and in adults, respectively):Congenital defects of phagocyte number or function (in children and in adults, respectively): 4 (3 + 1) (cyclic neutropenia: 2, CGD: 2)Diseases of immune dysregulation (in children and in adults, respectively): 7 (6 + 1) (autoimmune lymphoproliferative syndrome 5 (4 + 1), SAP deficiency 1 (1 + 0), familial hemophagocytic lymphohistiocytosis 1 (1 + 0).Congenital asplenia: 1 (1 + 0). Bold values indicate a statistically significant value.

## Methods

In order to identify IEI patients infected with SARS-CoV-2, a questionnaire was developed. It included questions about;

- Demographic data, the age of IEI, the type of deficiency, and its treatment and comorbidities;
- The age of SARS-CoV-2 infection and the reason and method of carrying out diagnostic tests to detect the infection;
- Symptoms, treatment, and complications;

The questionnaire was sent to clinical immunologists working in immunological centers in Poland. The mainstay for the diagnosis of COVID-19 was reverse transcription polymerase chain reaction (RT-PCR) tests, antigen tests, and/or the detection of IgM and/or IgG antibodies in people who had never been vaccinated against SARS-CoV-2. As the patients came from various centers in Poland, the tests were carried out with the use of the sets of different companies, but all of them had the required certifications.

The following analysis was made:

- Reason for testing for SARS-CoV-2 infection (before planned hospitalization, for epidemiological reasons—contact with the patient, clinical symptoms, other), month of infection (from 1 March 2020 to 30 April 2021);
- Clinical symptoms, especially fever, cough, the loss of smell and/or taste, pneumonia; the duration or recurrent nature of infection was analyzed (the diagnosis of a prolonged or relapsed form); the severity of the clinical course was defined;
- Acute complications, particularly respiratory failure, thromboembolic complications,
- “cytokine storm”, death; delayed complications, including the development of a multisystem inflammatory syndrome temporarily associated with SARS-CoV-2 in children, as well as progressive or chronic complications;
- Treatment: symptomatic, antibiotic therapy, remdesivir, convalescent plasma, passive oxygen therapy, mechanical ventilation.

The severity of the clinical course was defined as follows:

- Asymptomatic—no clinical symptoms, a positive result of the PCR or antigen test or the detection of IgM and/or IgG antibodies;
- Mildly symptomatic—clinical symptoms that resolved spontaneously, possible symptomatic treatment, without acute complications, no indications for treatment typical for COVID-19;
- Moderate—hospitalization, COVID-19-specific treatment and antibiotic therapy required (bacterial superinfection suspected), pneumonia with normal saturation, without serious acute complications;
- Severe—hospitalization and COVID-19-specific treatment needed, acute complications requiring intervention, a prolonged or recurrent course of the infection, chronic complications.

All the above parameters were compared both in the pediatric and adult groups.

The normality of the observed values was tested using the Shapiro–Wilk test. For the continuous variables, with non-normal distributions, the median (minimum to maximum) was used. Continuous variables were analyzed using the Mann–Whitney U test. Categorical variables were analyzed using the chi-square test or Fisher exact test. For all data analyses, differences were considered statistically significant when p < 0.05. Statistical analysis was performed using the STATISTICA software (TIBCO Software Inc. Palo Alto, CA, USA), version 13.

The conducted research was approved by the Bioethics Committee (No. KB 327/2022).

## Results

From March 2020 to the end of April 2021, COVID-19 was detected in 155 patients diagnosed with IEI. In the same period of time, 2,792,148 people (1) fell ill with COVID-19 in the general population in Poland.

### Characteristics of the group

Predominantly antibody deficiency (PAD) dominated in the whole group (105/155; 67.7%) but significantly more often in adults compared to children (94% vs. 53.3%, respectively; p <0.001). In the group of adult patients, common variable immunodeficiency (CVID) and X-linked agammaglobulinemia (XLA) were the most frequently diagnosed (52% vs. 16%, respectively), whereas 39% of the pediatric population were diagnosed with antibody deficiencies other than CVID and XLA. Moreover, in the pediatric group, among other diagnoses, severe combined immunodeficiencies (SCIDs) and combined immunodeficiencies (CIDs), including syndromic ones, constituted 24.8%. All patients with SCID (four children) were at least 6 months after allogeneic–hematopoietic cell transplantation (HCT). IEI from the group of autoinflammatory syndromes were diagnosed in 12% of pediatric patients. The most common IEI groups are presented in **Table 1**. A detailed distribution of diagnoses is provided in the form of footnotes under **Table 1**.

The most common treatment of the underlying disease in the IEI group was human immunoglobulin replacement therapy (IgRT): in the entire group in 105 patients (67.7%), significantly more often in adults compared to children (86% vs. 59%, respectively; p < 0.001) (**Table 1**).

At least one comorbidity was diagnosed in 79 patients (51%) in the entire group and two or more in 50 patients (32.3%). The incidence of comorbidities in IEI patients was significantly more common in adults than in children (56% vs. 21%, respectively; p < 0.001) (**Table 1**). Among patients with comorbidities, 40 people (25.8%) had immune diseases, including immune cytopenia, other autoimmune diseases, bronchial asthma, and other allergies. Non-immunological diseases, including neurological and psychiatric problems, chronic infections, and non-immunological gastroenterological diseases and others were present in 60 patients (38.7%). Due to the observed multiple morbidity, one patient could have one or more immunological and non-immunological comorbidities. A detailed list of comorbidities is presented in **Table 2**.

**Table 2:** Analysis of comorbidities in the presented group of patients with inborn errors of immunity (IEI), divided into immunological and non-immunological.

Feature	Whole group N (%)	Children N (%)	Adults N (%)	p-value
	155 (100%)	105 (67.7%)	50 (32.3%)	
Bronchial Asthma	15 (9.7 %)	11 (10.5 %)	4 (8.0 %)	0.626
Cytopenias	11 (7.1 %)	3 (2.9 %)	8 (16.0 %)	**0.003**
Other autoimmune	14 (9.0%)	8 (7.6%)	6 (12.0%)	0.27
Allergy	10 (6.5 %)	8 (7.6 %)	2 (4.0 %)	0.391
Cardiac defects	7 (4.5 %)	7 (6.7 %)	0 (0.0 %)	0.062
Gastrointestinal diseases	11 (7.1 %)	6 (5.7 %)	5 (10.0 %)	0.331
Obesity	7 (4.5 %)	2 (1.9 %)	5 (10.0 %)	**0.023**
Pulmonary (bronchiectasis, GLILD)	8 (5.2%)	4 (3.8%)	4 (8.0%)	0.232
Hypertension	7 (4.5 %)	0 (0.0 %)	7 (14.0 %)	**< 0.001**
Chronic infections	11 (7.1 %)	3 (2.9 %)	8 (16.0 %)	**0.003**
Prematurity	2 (1.3 %)	2 (1.9 %)	0 (0.0 %)	0.326
Diabetes	3 (1.9 %)	1 (1.0 %)	2 (4.0 %)	0.198
Cancers*	4 (2.6%)	0 (0.0%)	4 (8.0%)	**0.01**
Neurological/psychiatric diseases	14 (9.0 %)	8 (7.6 %)	6 (12.0 %)	0.374

*One newly diagnosed and three in anamnesis, without active anticancer treatment. Bold values indicate a statisticallysignificant value.

### Diagnosis of COVID-19 in the group

**Figure 1:**
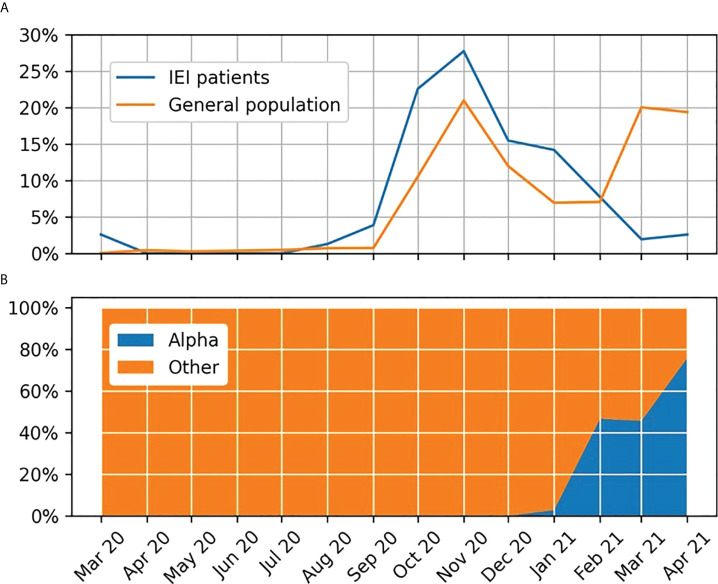
**(A)** Percentage of monthly COVID-19 diagnoses in the Polish population and among inborn errors of immunity patients. **(B)** Variants of the SARS-CoV-2 virus reported in the analyzed period in Poland.

In the study group, COVID-19 was occasionally diagnosed until August 2020 (3.2%). The largest number of patients with IEI became infected in October and November 2020 (22.6% and 27.8%, respectively), which was correlated with the incidence of infections in the general population in Poland. In February 2021, the increase in infections in patients with IEI was observed 1 month earlier than in the general population (**Figure 1A**). Unfortunately, in Poland, SARS-CoV-2 virus variants were not identified or reported as “different” in the first months of the pandemic. Single alpha variants were reported between October 2020 and January 2021. The analyses of SARS-CoV-2 variants began on a larger scale from February 2021, when the alpha variant was definitely dominant in Poland (8) (**Figure 1B**)

For the entire group, the age of patients with IEI at the time of SARS-CoV-2 infection ranged from 0.5 to 82 years, with a median of 13 years, in the pediatric population from 0.5 to 17 years, with a median of 8.5 years, and in adults from 18 to 82 years with a median of 42.5 (**Table 3**).

**Table 3:** The results of research on COVID-19 infection in patients with IEI.

Feature	Whole group N (%)	Children N (%)	Adults N (%)	p-value
	155 (100%)	105 (67.7%)	50 (32.3%)	
**Age at the diagnosis of COVID-19:**				
Min/max/average/median/standard deviation	0.5/82/19.7/13/18.5	0.5/17/8.7/8.5/4.7	19/82/42.5/42.5/15.4	<0.001
Female n (%)	61/155 (39.4%)	38/105 (36.2%)	23/50 (46.0%)	0.294
**Diagnosis of COVID-19:**				
until 31/08/2020 01/09/20–30/04/21	6/155 (3.9%) 149/155 (96.1%)	5/105 (4.8%) 100/105 (95.2%)	1/50 (2.0%) 49/50 (98.0%)	0.665 0.665
**Reason for testing for SARS-CoV-2:**				
Symptoms Other	124/155 (80%) 31/155 (20%)	78/105 (74.3%) 27/105 (25,7%)	46/50 (92%) 4/50 (8%)	**0.01**
**Diagnostic method:**				
PCR Ag Ab	105/155 (67.7%) 5/155 (3.2%) 54/155 (34.8%)	58/105 (55.2%) 5/105 (4.8%) 51/105 (48.6%)	47/50 (94.0%) 0/50 (0.0%) 3/50 (6.0%)	**<0.001** 0.176 **<0.001**
**Clinical symptoms:**				
Fever Cough Smell impairment Taste impairment Other	84/155 (54.2%) 57/155 (36.8%) 16/155 (10.3%) 21/155 (13.5%) 94/155 (60.6%)	45/105 (42.9%) 26/105 (24.8%) 5/105 (4.8%) 9/105 (8.6%) 61/105 (58.1%)	39/50 (78.0%) 31/50 (62.0%) 11/50 (22.0%) 12/50 (24.0%) 33/50 (66.0%)	**<0.001** **<0.001** **0.003** **0.012** 0.383
**Course:**				
Asymptomatic Mildly symptomatic Moderate Severe	31/155 (20.0%) 84/155 (54.2%) 21/155 (13.5%) 19/155 (12.3%)	27/105 (25.7%) 66/105 (62.9%) 10/105 (9.5%) 2/105 (1.9%)	4/50 (8.0%) 18/50 (36.0%) 11/50 (22.0%) 17/50 (34.0%)	**0.01** **0.002** **0.045** **<0.001**
**Treatment:**				
Antibiotic therapy Remdesivir Convalescent plasma Passive oxygen therapy Mechanical ventilation Other	45/155 (29.0%) 12/155 (7.7%) 12/155 (7.7%) 14/155 (9.0%) 9/155 (5.8%) 34/155 (21.9%)	15/105 (14.3%) 1/105 (1.0%) 2/105 (1.9%) 2/105 (1.9%) 0/105 (0.0%) 22/105 (21.0%)	30/50 (60%) 11/50 (22.0%) 10/50 (20.0%) 12/50 (24.0%) 9/50 (18.0%) 12/50 (24.0%)	**<0.001** **<0.001** **<0.001** **<0.001** **<0.001** 0.682

Bold values indicate a statistically significant value.

The reason for testing for SARS-CoV-2 infection in almost all adults (46/50; 92%) was the occurrence of the symptoms of the infection. In the pediatric group, the symptoms were a less frequent indication for testing than in adults (78/105; 74.3%). Approximately 25% of children and sporadic adults were tested due to epidemiological reasons (e.g., contact with SARS-CoV-2 in the school or home), including tests before or on admission to hospital (4/50; 8% and 27/105; 25.7%). In the presented group, COVID-19 was most frequently diagnosed using nasopharyngeal swab RT-PCR testing (67.7%), significantly more often in adults than in children (94% vs. 55.2%; p < 0.001). In children, the diagnosis was relatively frequently made based on anti-SARS-CoV-2 antibody titers (48.6%) (**Table 3**).

### The course and treatment of COVID-19 in the group

The symptoms typical for SARS-CoV-2 that occurred significantly more often in adults compared to children were fever: 76% vs. 42.9%; p <0.001, cough: 60% vs. 24.8%; p <0.001, loss/disorders of smell: 20% vs. 4.8%; p = 0.006, and taste disorders: 22% vs. 8.6%; p = 0.037.

Other symptoms of infection (sore throat, runny nose, headache, abdominal pain, vomiting, diarrhea, chest pain, dyspnea, and enlarged lymph nodes) were also slightly more often reported by adult patients (66% vs. 58.1%; p = 0.383) (**Table 3**).

The asymptomatic course of COVID-19 was demonstrated in 20% of patients in the entire study group, significantly more often in children than in adults (25.7% vs. 8%; p = 0.01). The mildly symptomatic course was identified in 54.8% of the whole group, significantly more often in children compared to adults (62.9% vs. 38%; p = 0.005). The moderate course concerned 13.5% of all patients, significantly more often adults than children (22% vs. 9.5%; p = 0.045).

Severe COVID-19 concerned 11.6% of patients, almost exclusively adults (32% vs. 1.9%; p<0.001) (**Table 3**).

Antibiotic therapy was the most common treatment of COVID-19 (in 29% of the entire group), significantly more often in adults than in children (60% vs. 14.3%; p<0.001). COVID-19-specific therapy was used almost exclusively in adults: remdesivir in 20% and convalescent plasma in 18%. These therapies were used in 1% and 1.9% of children, respectively. Similarly, passive oxygen therapy was used in 11% of adults and 1.9% of children. Mechanical ventilation was used only in 16% of adults (**Table 3**).

### Complications of COVID-19

In the whole group, complications occurred in 14.2% of patients, significantly more often in adults than in children (30% vs. 6.7%; p <0.001). Regardless of age, among 22 patients with complications, as many as 9 (40.9%) were diagnosed with the coexistence of immunological and non-immunological comorbidities. Among 133 patients without the complications of COVID-19, only 18 (13.2%) had both immunological and non-immunological complications. The difference is statistically significant (p = 0.002). On the other hand, the lack of comorbidities significantly more often affected people without complications: 71/133 (53.4%) vs. 5/22 (22.7%) with complications (p = 0.008).

The following complications were observed in adults: respiratory failure in 14%, pulmonary complications (bacterial pneumonia as superinfection, pneumothorax, pulmonary fibrosis) in 12%, other complications in 8% (one case each: fever prolonged to 12 weeks, chronic urticaria, worsening of comorbidities of IEI, and sepsis).

Other complications occurred in individual children. The multisystem inflammatory syndrome in children (MIS-C) was diagnosed in two patients (2/105; 1.9%): in a 1-year-old girl with SCID within >100 days from HCT and in a 6-month-old girl with hypogammaglobulinemia that eventually turned out to be transient. Both girls were treated with high-dose immunoglobulins and steroids. The younger child developed giant aneurysms in the coronary arteries, requiring constant anticoagulation therapy. Decreased physical activity was observed in a 14-year-old boy with CVID. In the remaining five pediatric patients, sporadic complications such as behavioral disturbances and depression (two patients), one case each of diarrhea and long-lasting dysgeusia were reported. These complications were related to patients diagnosed with severe hypogammaglobulinemia with normal B cells and secondary causes excluded.

There were no thromboembolic complications in the presented group.

In the entire group, six adult individuals (3.9%) died due to COVID-19 (6/50; 12%): three men aged 34, 39, and 66 and three women aged 51, 53, and 62. Four out of six patients who died were diagnosed with CVID (4/26; 15.4%), one with XLA (1/8; 12.5%) and one with selective immunoglobulin A deficiency (sIgAD). In the presented study, sIgAD is included in the “other humoral defects” group (1/13; 7.7%). All patients who died were diagnosed with two or more comorbidities of IEI (6/28; 21.4% of adults with at least two comorbidities) (**Tables 3**, **4**). Four of the deceased patients were diagnosed with both immunological and non-immunological comorbidities. They were:

**Table 4:** Analysis of complications and deaths depending on selected diagnoses and comorbidity.

	Whole group	Children	Adults	p-value
**Severe course (complications and/or death)**				
**All diagnoses/any event:**	22/155 (14.2%)	7/105 (6.7%)	15/50 (30.0%)	**<0.001**
**Diagnosis:**				
Humoral defect XLA CVID	21/103 (20.4%) 4/10 (40.0%) 9/39 (23.1%)	6/56 (10.7%) 0/2 (0.0%) 1/13 (7.7%)	15/47 (31.9%) 4/8 (50.0%) 8/26 (30.8%)	**0.013** 0.467 0.225
**≥2 comorbidities**	14/49 (28.6%)	1/21 (4.8%)	13/28 (46.4%)	**0.001**
**Type of complications:**				
Respiratory failure Pulmonary complications Other	8/155 (5.2%) 6/155 (3.9%) 14/155 (9.0%)	0/105 (0.0%) 0/105 (0.0%) 7/105 (6.7%)	8/50 (16.0%) 6/50 (12.0%) 7/50 (14.0%)	<0.001 <0.001 0.146
**Death**				
**All diagnoses:** Humoral defect XLA CVID	6/155 (3.9%) 6/103 (5.8%) 1/10 (10.0%) 4/39 (10.3%)	0/105 (0.0%) 0/56 (0.0%) 0/2 (0.0%) 0/13 (0.0%)	6/50 (12.0%) 6/47 (12.8%) 1/8 (12.5%) 4/26 (15.4%)	**<0.001** **0.008** 1.0 0.281
**≥2 comorbidities:**	6/49 (12.2%)	0/21 (0.0%)	6/28 (21.4%)	**0.031**

Bold values indicate a statistically significant value.

- Patient 1: bronchial asthma, chronic sensorimotor polyneuropathy, diabetes, arterial hypertension, obesity, hyperlipidemia, condition after lung abscess treatment;
- Patient 2: inflammatory bowel disease, malabsorption syndrome, cachexia;
- Patient 3: chronic urticaria, iron deficiency anemia, chronic esophagitis, pituitary tumor, hyperprolactinemia;
- Patient 4: autoimmune thyroiditis, sarcoidosis, hypertension, chronic sinusitis.

One patient deceased was diagnosed with immune-related comorbidities (bronchial asthma and Hashimoto thyroiditis). The last deceased patient suffered from non-immune accompanying diseases: chronic otitis media, audiological complications, hyperhomocysteinemia, and profound vitamin D deficiency (**Table 2**).

## Discussion

Since the beginning of the pandemic, there have been many publications on COVID-19 in IEI patients, which include the descriptions of single or several cases, reports of SARS-CoV-2 infections identified in individual centers or countries, and presentations of data collected as part of international projects (6, 7, 9–11). Some of them were meta-analyses (4, 12). Much of the information was related to adult patients, while only individual publications exclusively concerned children (13–15). Our own analysis shows a relatively large group of patients with IEI (155 people) diagnosed with SARS-CoV-2 infection. The study group was dominated by children (67.7%). This, however, does not prove a higher incidence of SARS-CoV-2 infections in the pediatric population with IEI in Poland. In the authors’ opinion, it is the result of more effective identification of children with IEI and COVID-19 than in the corresponding adult population. In Poland, the network of pediatric care centers providing care for patients with IEI is better developed compared to internal medicine centers. The lack of an effective, nationwide system of registration of patients with IEI does not make the task easier.

According to the summaries of the ESID registry from 2014, IEI with predominantly antibody defects were diagnosed in 56.7% of the reported people (16). The distribution of individual IEI diagnoses in the study group was typical for European populations, in which IEI with predominantly antibody defects prevail. However, the distribution may be different depending on the population (17–20).

The reason for testing for SARS-CoV-2 infection was slightly different in adults compared to children. In adults, the indication concerned almost exclusively the symptoms of the disease (92%), while in approximately one-fourth of children, the tests were conducted based on epidemiological premises or before planned admission to hospitals. This was partially due to the fact that most of the adult patients had the symptoms of infection, while in approximately 25% of children, the course of the infection was asymptomatic. In Poland, many patients from the pediatric group were tested at the request and expense of their parents. Therefore, in this population, cheaper serological tests were performed significantly more often than in adults (48.6% vs. 6%). In 94% of adults, the diagnosis was made on the basis of PCR tests, which are considered the gold standard in diagnostics, especially in patients with predominantly antibody defects (12).

In the presented group, all symptoms considered typical for COVID-19 occurred significantly more often in adults. The most persistent ones were fever and cough. Taste and smell disorders were rare in children. The other less characteristic symptoms of COVID-19 were also more common in adults than in children. Similar results were obtained in other studies, although the typical comparative analyses of clinical picture pediatric population and in adults were not found in the available literature (7, 12, 21).

At the beginning of the pandemic, IEI patients appeared to be at a high risk of developing COVID-19. However, it soon turned out to be entirely true. One of the largest meta-analyses of 649 patients with IEI and COVID-19 showed that the diagnosis of IEI is generally not a risk factor for a severe course of the disease. However, individual IEI groups may be associated with a higher risk of a severe course and death. These include combined immunodeficiencies, immune dysregulation syndromes, and certain defects of the innate immune system, especially those related to the type I interferon–associated immune response (4). Humoral immune defects definitely dominated in the group presented here. It is not surprising then that in 74.8% of patients the course of COVID-19 was asymptomatic or mildly symptomatic. In an interesting summary of publications on COVID-19 in patients with IEI by Quinti et al., the course was asymptomatic or mildly symptomatic in a smaller percentage of patients (48%), even that the most of the analyzed publications concerned adults. Our data limited to adults only compared with Quinti’s seem to be almost identical (46% vs. 48%) (12). Different results were obtained in a Czech study of 81 patients with IEI and COVID-19. In this group, the asymptomatic course concerned almost the same percentage of patients as in presented analysis (21% vs. 20%), but the risk of seriously severe COVID-19 was 2.3 times higher than in the general population. However, taking into account the fact that the mean age of the population was >42 years while in our study—19 years), it can be assumed that the vast majority of the analysis concerned elderly adults, which is itself a risk factor (21).

It was observed very early that the course of SARS-CoV-2 infection in adults is much more serious than in children (5). Own study confirmed that this trend also applies to IEI patients. The vast majority of IEI children were asymptomatic or mildly symptomatic (88.6%), while 56% of adults presented moderate-to-severe symptoms. Other researchers (7, 10, 11, 14, 22, 23) made similar observations. Delavari et al. observed a severe course of COVID-19 in children (6). However, it is worthy to note a different spectrum of IEI diagnoses in reported Iranian population. In total, 10 out of 19 described patients were children with combined immunodeficiencies before hematopoietic stem cell transplantation. Predominantly antibody defects occurred only in four patients. It is not surprising that more than 56% of patients in this group had a severe course of SARS-CoV-2 infection (6).

No generally accepted standard of treatment for patients with IEI and COVID-19 has been developed so far. Only single reports described the efficacy of convalescent plasma or specific monoclonal antibody cocktails against the viral spike protein in combination with antiviral drugs (remdesivir) at an early stage of SARS-CoV-2 infection. However, most often, patients with IEI and COVID-19 were treated in line with local guidelines (12, 21, 24). The treatment used in hospitalized patients is often the subject of analysis in the available literature, but there are usually no data on home therapy (6, 7, 12, 21). In the presented group, systemic antibiotic therapy was the most common therapeutic option used in symptomatic patients. In the pediatric group, apart from symptomatic treatment, it was almost the only therapy used and concerned 14.3% of patients. Antibiotic therapy was significantly more often used in adults (60%), which additionally emphasizes a more serious course of the disease in this population. The need for antibiotic therapy in some children and most adult patients with IEI and COVID-19 was demonstrated by other authors (6, 7, 12, 24). The remaining therapies were used almost exclusively in adults. At that time, Polish patients did not have access to monoclonal antibodies specifically blocking the SARS-CoV-2 spike protein.

One of the most important aspects of caring for patients with IEI and COVID-19 is the analysis of complications and deaths. It should constitute the basis for defining the risk factors of a serious course and an attempt to develop the principles of prophylaxis and treatment, especially at the beginning of the disease, which would prevent it from worsening. In the available literature, there are little unambiguous data indicating specific risk factors related to a type of immunodeficiency, the comorbidities of IEI, and the treatment used. IEI groups were defined, which are clearly associated with a high risk of a poor prognosis, as mentioned above.

Among people diagnosed with SCID, CID, immune dysregulation syndromes, and type I interferon defects, the risk of complications and death seems to be independent of age and may also affect children (4, 12). In the presented pediatric group, no deaths were reported despite the diagnosis of combined immunodeficiencies and autoinflammatory syndromes in 37% of patients. However, two patients (1.9%) were diagnosed with MIS-C. The obtained results may indicate a much-higher risk of this complication than in the general pediatric population. In the American analysis of the epidemiology of MIS-C in the population up to 21 years of age, among white people, it was estimated that this diagnosis concerns an average of 110 per 1 million people infected with SARS-CoV-2, which is 0.011% (25). In the early stage of the pandemic, there were reports that the risk of complications and death is higher in patients with CVID compared to patients with XLA (9). The studies conducted by Cohen in the group of patients with CVID did not confirm a higher risk of a serious course of COVID-19 in this population (26). The currently available meta-analyses show that the risk of a complicated course of COVID-19 is the same in the entire IEI group with predominantly antibody defects as in the separated subgroup of CVID or XLA (14% each), and the risk of death is very similar (8%, 9%, and 8%) (4). In the analysis of Bucciol et al., the pediatric and adult subgroups were not separated, which allows only for their comparison with our own results obtained for the entire cohort: for all IEI, the percentage of patients with complications and/or death was 14.2%, for CVID 23.1%, and for XLA 40%. The analysis of only deaths compared to the results presented by Bucciol et al. shows that in the entire analyzed group, the percentage was lower (3.9%), while it was very similar for patients with CVID (10.3%) and XLA (10%) (4). In the study by Sheidel et al., in the group of patients with both CVID and XLA, there were only adults; hence, the data were compared with our own results obtained in patients >18 years of age. Mortality associated with SARS-CoV-2 infection in patients with CVID in the British study was slightly higher than in our own study (18.3% and 15.4%, respectively) and significantly lower in patients with XLA (7.7% vs. 12.5%, respectively). However, in our own study, there were only 8 adult patients diagnosed with XLA and 26 in the British group (24).

Our study indicates a significant role of comorbidities in the severe course in COVID-19 and/or fatal outcome in IEI patients (complicated course including death—46.4%, death only—21. 4%). This is consistent with the data on the general population, as well as many studies on IEI patients (5, 7, 24). The recently published study by Shields et al. showed that an additional risk factor for serious complications and death from COVID-19 in people with IEI is lymphopenia diagnosed in patients before infection (24). This parameter was not analyzed in our study.

Patients with IEI, especially those predisposing to the severe course of COVID-19, as well as those with comorbidities, have indications for preventive vaccination with the use of a booster dose (in Poland, ≥5 years of age). It is also a population in which an inadequate vaccine response is possible. Therefore, in the periods of special epidemiological threat and special health situations, it is recommended to use pre-exposure prophylaxis with the use of long-acting monoclonal antibodies (≥12 years of age), as well as the early implementation of anti-SARS-CoV-2 treatment. Recommendations regarding postexposure prophylaxis change, depending on the effectiveness of the available drugs in relation to the dominant variants of the SARS-CoV-2 virus (27).

## Summary

The present study confirms that in patients with severe deficits of humoral immunity (CVID and XLA), this risk for a severe course of COVID-19 increases significantly with age. An important aggravating factor is comorbidity, especially in people >18 years of age. In children, regardless of the type of IEI, the risk of a severe course of COVID-19 is very low. However, the risk of developing MIS-C might be higher than in the general population—that requires further testing in larger groups of patients vs. general population. Therefore, the vaccination of both adults and children should be promoted. What will be their effectiveness in prevention and the impact on the course of the disease remain to be seen. It seems that our study might justify the recommendation to use SARS-CoV-2-specific post-exposure prophylaxis and early treatment with antiviral drugs, SARS-CoV-2-specific monoclonal antibodies, or convalescent plasma in adults, especially those with diagnosed CVID and XLA and comorbidities.

## Strengths and limitations of the study

The lack of Polish registry makes it impossible to accurately determine the number of patients with IEI. In addition, patients with COVID-19 were not referred directly to immunologists but to primary care physicians and hospitals treating COVID-19. Moreover, data were not obtained from every center treating patients with IEI.

## Data availability statement

The raw data supporting the conclusions of this article will be made available by the authors, without undue reservation.

## Ethics statement

The studies involving human participants were reviewed and approved by The Bioethics Committee, Collegium Medicum Bydgoszcz, Nicolaus Copernicus University Toruń, No. KB 327/2022. Written informed consent to participate in this study was provided by the participants’ legal guardian/next of kin.

## Author contributions

SK, MZ, and MP contributed to conception and design of the study, supervised the study process, and critically analyzed the manuscript for important intellectual content. SK, MZ, EG, RB, EB-S, MC, ME, EH-P, NK, AL-U, MM-T, AM-B, MM, KN-B, KP-Ś, AP-N, JRo, JRe, IR, AS-G, MS, HS, JS-G, AS-P, ST, EW-S, BW-K, KZ, and MP organized the database. SK wrote the original version of the manuscript. SK and ME reviewed the literature and drafted the manuscript. All authors contributed to manuscript revision and read and approved the submitted version.

## Funding

The publication was financed by Uniwersytet Mikołaja Kopernika in Toruń (Interdisciplinary Innovation in Personalized Medicine).

## Conflict of interest

The authors declare that the research was conducted in the absence of any commercial or financial relationships that could be construed as a potential conflict of interest.

## Publisher’s note

All claims expressed in this article are solely those of the authors and do not necessarily represent those of their affiliated organizations, or those of the publisher, the editors and the reviewers. Any product that may be evaluated in this article, or claim that may be made by its manufacturer, is not guaranteed or endorsed by the publisher.
